# Correction: Siu et al. Hexokinase 2 Regulates Ovarian Cancer Cell Migration, Invasion and Stemness via FAK/ERK1/2/MMP9/NANOG/SOX9 Signaling Cascades. *Cancers* 2019, *11*, 813

**DOI:** 10.3390/cancers16213633

**Published:** 2024-10-28

**Authors:** Michelle K. Y. Siu, Yu-Xin Jiang, Jing-Jing Wang, Thomas H. Y. Leung, Chae Young Han, Benjamin K. Tsang, Annie N. Y. Cheung, Hextan Y. S. Ngan, Karen K. L. Chan

**Affiliations:** 1 Department of Obstetrics and Gynecology, University of Hong Kong, Hong Kong, China; mkysiu@gmail.com (M.K.Y.S.); yuxin_jiang2012@126.com (Y.-X.J.); wjj01947@connect.hku.hk (J.-J.W.); thyl@hku.hk (T.H.Y.L.); hysngan@hku.hk (H.Y.S.N.); 2 Department of Obstetrics and Gynecology and Cellular and Molecular Medicine, University of Ottawa, Ottawa, ON K1H 8L6, Canada; lemon7time@gmail.com (C.Y.H.); btsang@ohri.c (B.K.T.); 3 Chronic Disease Program, Ottawa Hospital Research Institute, Ottawa, ON K1H 8L6, Canada; 4 State Key Laboratory of Quality Research in Chinese Medicine, Macau Institute for Applied Research in Medicine and Health, Macau University of Science and Technology, Macao, China; 5 Department of Pathology, University of Hong Kong, Hong Kong, China; anycheun@pathology.hku.hk

In the original publication [1], there was a mistake in Figure 1D as published. The loading control in Figure 1D was unintentionally misused from our previously published data from the same batch of samples. The experiment in Figure 1D was repeated. The legend remains unchanged. The corrected Figure 1 appears below.

In the text, a typo (TOV112D instead of OC316) was observed. A correction has been made in Section 2. Results, 2.1. Overexpression of HK2 Correlates with Ovarian Cancer Metastasis and Patient Prognosis, the last sentence: By western blot analysis, we found an up-regulation of HK2 protein expression in ovarian cancer cell lines (OVCAR-3, OVCA429, OVCA433, ES-2, TOV21G, TOV112D, A2780S, and A2780CP), compared to normal ovarian epithelial cell lines (HOSE 6-3 and HOSE 11-12) (Figure 1D).

In the supplementary materials, we have included Figure S5, which displays the original blot forWestern blot analyses in Figure 1D; Figure S6, which displays the density readings and intensity ratios for each Western blot band presented in Figures 2–6.

The authors apologize for any inconvenience caused and state that the scientific conclusions are unaffected. This correction was approved by the Academic Editor. The original publication has also been updated.

## Figures and Tables

**Figure 1:**
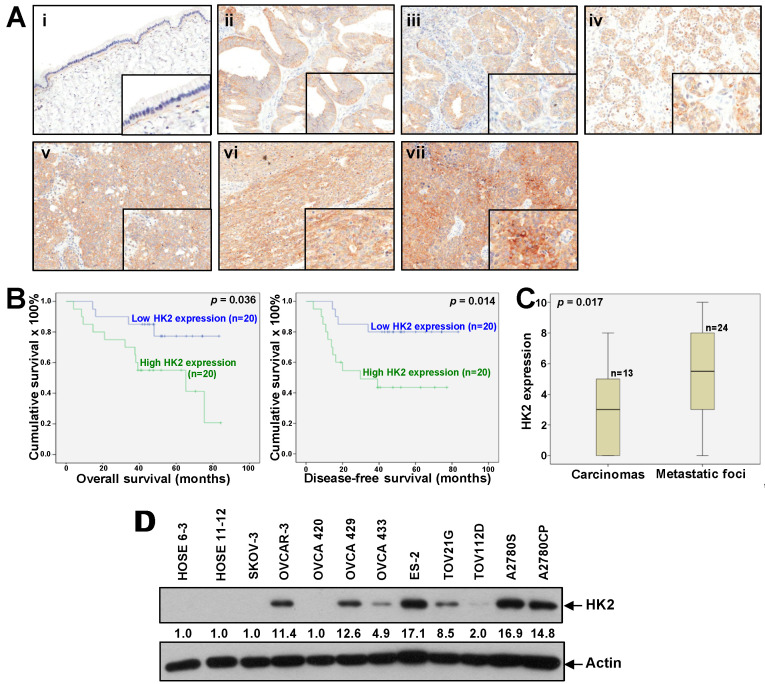
Up-regulated HK2 in ovarian cancer is linked to tumor metastasis and poor survival. (**A**) Immunohistochemical staining of HK2 in mucinous benign cystadenoma (**i**); mucinous (**ii**), endometrioid (**iii**), and clear cell (**iv**) carcinomas; primary serous carcinomas (**v**); and matched metastatic foci (**vi**) and (**vii**). Magnification: 20×. The insets highlight regions with higher magnification. (**B**) Kaplan–Meier overall (left panel) and disease-free (right panel) survival curves for ovarian cancer patients with low and high HK2 expression levels (cut-off at mean). (**C**) HK2 immuno-scoring in primary carcinomas and corresponding metastatic foci. (**D**) HK2 protein expression in normal ovarian epithelial cell lines (HOSE) and ovarian cancer cell lines as assessed by immunoblot analysis. Densitometric analysis is shown normalized to Actin.
